# The causal associations between growth factors and constipation: a two-sample Mendelian randomization study

**DOI:** 10.3389/fphys.2023.1204146

**Published:** 2023-07-12

**Authors:** Jiachen Wang, Mingyi Yang, Ke Xu, Xianjie Wan, Jiale Xie, Hui Yu, Jiaxin Fang, Zehua Wang, Peng Xu

**Affiliations:** ^1^ Department of Joint Surgery, HongHui Hospital, Xi’an Jiaotong University, Xi’an, Shaanxi, China; ^2^ Department of Clinical Medicine, Xi’an Jiaotong University, Xi’an, Shaanxi, China

**Keywords:** cell growth factors, constipation, genome-wide association study, two-sample Mendelian randomization, single-nucleotide polymorphisms

## Abstract

**Introduction:** Certain growth factors (GFs) are associated with constipation, but few studies has analyzed the causal associations between the two. Therefore, this study used two-sample Mendelian randomization (MR) to systematically analyze the causal associations between GF levels and constipation based on data from genome-wide association studies (GWAS).

**Methods:** Both GF and constipation data were obtained from European populations. GFs, as an exposure variable, were obtained from a genetic map of the human plasma proteome containing 3,301 samples, another GWAS dataset on 90 circulating proteins containing 30,931 samples, and a GWAS dataset containing 3,788 samples. Constipation, as an outcome variable, was obtained from the FinnGen project containing 26,919 cases and 282,235 controls and another UK Biobank dataset containing 3,328 cases and 459,682 controls. Single-nucleotide polymorphisms strongly associated with GFs were regarded as instrumental variables. Inverse-variance weighting, MR–Egger regression, weight median, simple mode, and weight mode methods were used to determine genetic associations. Cochran’s Q test, Egger intercept, and Mendelian Randomization Pleiotropy RESidual Sum and Outlier tests were used to analyze sensitivity.

**Results:** The IVW analysis based on FinnGen showed that NGFI-A-binding protein 2 and vascular endothelial growth factor receptor 2 were inversely associated with constipation, and that fibroblast growth factor 7 and transforming growth factor beta receptor II levels were positively associated with constipation. The IVW analysis based on UK Biobank showed that proheparin-binding epidermal growth factor, platelet-derived growth factor AA, and vascular endothelial growth factor_121_ were inversely associated with constipation.

**Conclusion:** This study showed that some GFs are genetically associated with the risk of constipation.

## 1 Introduction

Constipation is a common gastrointestinal disease, with an overall global prevalence of approximately 14% (Black and Ford, 2018); the prevalence in children and elderly people (over 60 years old) is approximately 9.5% and 33.5%, respectively (Forootan et al., 2018; Koppen et al., 2018). Constipation itself is not fatal, but chronic constipation can seriously affect the quality of life (Camilleri et al., 2017; Bharucha and Wald, 2019). Moreover, constipation significantly increases the incidence of coronary heart disease, ischemic stroke, and the all-cause mortality of the elderly population, bringing a heavy disease burden to the elderly population (Sumida et al., 2019). According to the Rome criteria, constipation is divided into functional constipation (FC), constipation-predominant irritable bowel syndrome (IBS), defecatory disorders (DDs), and opioid-induced constipation (OIC) (Mearin et al., 2016). FC is the most common type of constipation, accounting for approximately 60% of constipation cases (Palsson et al., 2020). It lacks an organic cause, and all organ structures or metabolism are normal (Ford et al., 2014). However, its pathogenesis remains unclear. Currently, the factors affecting pathogenesis of functional constipation include intestinal flora, bile excretion, lifestyle, and behavioral psychology (Black and Ford, 2018). IBS is a chronic intestinal disease with repeated abdominal pain and changes in bowel habits as the main symptoms, without abnormalities in the digestive system structure or biochemical indicators. It is categorized into diarrhea-based (IBS-D), constipation-based (IBS-C), or mixed (IBS-M) types (Saha, 2014; Hanning et al., 2021). The etiology is not completely clear and may be related to factors such as visceral sensitivity, gastrointestinal motility, gastrointestinal mucosal permeability, changes in intestinal microecology, and intestinal inflammation (Simrén and Tack, 2018; Bonetto et al., 2021). DD is mainly caused by damage to the rectum or the muscles that control rectal movement (such as the abdominal wall muscles, pelvic floor muscles, or anal sphincter) and is common in patients with rectal prolapse, prolapse, or intussusception (Nullens et al., 2012).

Growth factors (GFs) are a class of natural substances that stimulate cell growth and differentiation (Li et al., 2013b). According to their different functions, they can be roughly categorized into epidermal growth factor (EGF), fibroblast growth factor (FGF), nerve growth factor (NGF), and transforming growth factor (TGF) (Bafico and Aaronson, 2003). Some GFs are also immunomodulatory, which may cause constipation or IBS by affecting gastrointestinal inflammation development (Larson et al., 2020; Uranga et al., 2020); however, current evidence is still insufficient to support causal associations between GFs and constipation.

Mendelian randomization (MR) studies are statistical methods for testing causal associations between exposures on outcomes. It uses a random single-nucleotide polymorphism (SNP) distribution in the population as an instrumental variable (IV) and indirectly infers the causal association between exposure and outcomes by calculating the causal effect value of the IV on exposure and outcomes (Yang et al., 2022). Compared with traditional observational studies, MR studies have advantages, such as a large sample size and reduced influence of unknown confounding factors and reverse causality (Kamiza et al., 2022). Because of these advantages, MR has been widely used in studies in recent years, for example, MR has been used to determine the genetic causal associations between diabetes and cataract and peripheral vascular disease (Xiu et al., 2022; Zhang et al., 2022).

In this study, two-sample MR was used to analyze the causal association between various GFs (including their receptors) and constipation. Then, the GFs and related signaling pathways that caused constipation were screened to provide an insight into the constipation development mechanism. This study also provides clues for future research on therapeutic targets for constipation.

## 2 Materials and methods

### 2.1 Study design

**FIGURE 1:**
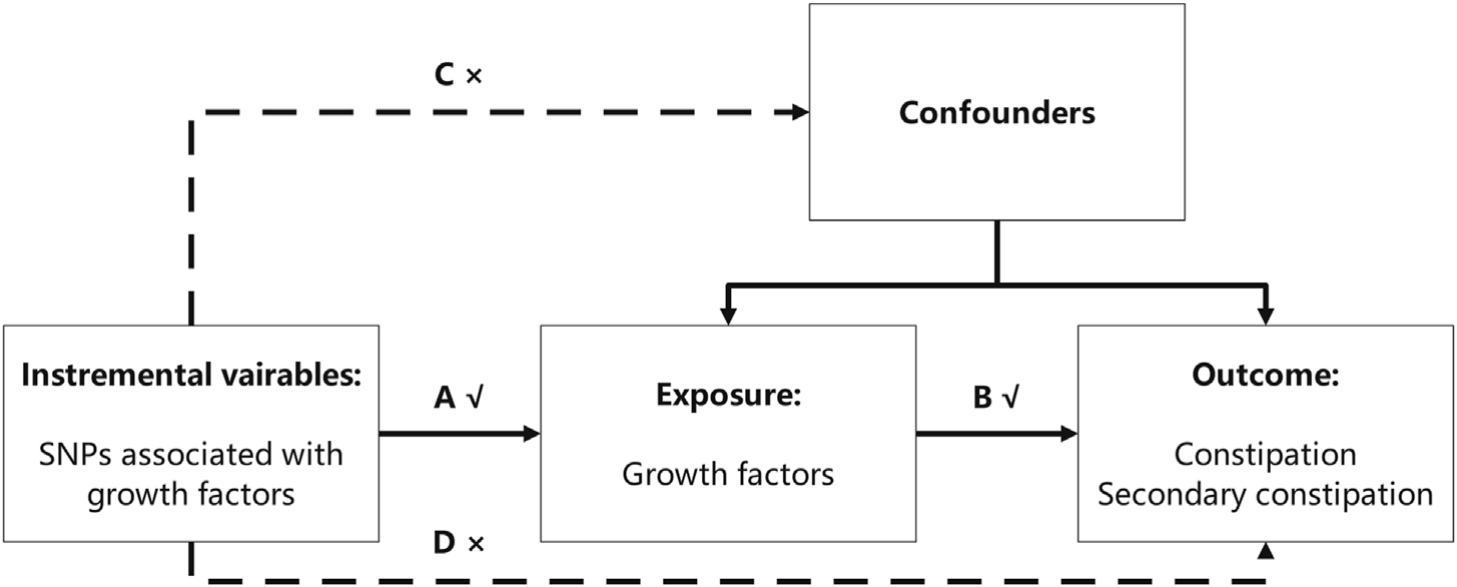
Three assumptions the MR analysis must meet. In this figure, the single-nucleotide polymorphisms (SNPs) strongly associated with growth factors were regarded as instrumental variables (IVs), and all the IVs should not affect the outcomes directly or through confounders (the dashed line and arrows “C” and “D” are not allowed) to guarantee the second assumption. The selected SNPs can only affect the outcomes with exposure (solid line and arrow “A” and “B” are permitted) to guarantee the third assumption.

This study aimed to explore the causal effect of GFs on constipation using the MR method. Two-sample MR requires non-overlapping samples from genome-wide association study (GWAS) pooled data for identifying the exposure and outcome variables. In this study, GFs and constipation were regarded as an exposure and an outcome, respectively. For MR analysis, three assumptions must be satisfied: first, the IVs choice must be strongly associated with exposure; second, the IVs should be independent of the observed or unobserved confounding factors; and third, the IVs should only affect the outcome variables via exposure factors (Lawlor et al., 2008) (Figure 1).

### 2.2 Data resources

The GFs were regarded as exposure, and the exposure data were obtained from three GWAS summary datasets obtained from the IEU OpenGWAS project, an open database (https://gwas.mrcieu.ac.uk/). The first GWAS data for GFs were obtained from a genetic map of the human plasma proteome. The study sample included 3,301 healthy blood donors from the INTERVAL study (European population), and 3,622 plasma proteins were quantified (Sun et al., 2018). The second GWAS data for GFs were extracted from another database on 90 circulating proteins identified from 30,931 European individuals (Folkersen et al., 2020). The third GWAS study for GFs aimed to link the genetic risk to disease endpoints using the human plasma proteome, and this study contains 3,788 European individuals (Suhre et al., 2017).

The outcome data were obtained from two GWAS pooled datasets. The first constipation GWAS summary statistics were obtained from FinnGen (https://r7.finngen.fi/). The results have been corrected for sex, age, top 10 principal components, genotype batch, and genetic kinship, and 26,919 cases and 282,235 controls were included (Kurki et al., 2023). Furthermore, this study also used UK Biobank GWAS datasets from the IEU OpenGWAS project, including primary diagnosis ICD10: constipation (3,328 cases and 459,682 controls) and primary diagnosis secondary ICD10: constipation (3,862 cases and 459,148 controls).

### 2.3 Instrumental variable selection

Under the genome-wide significance threshold (*p* < 5 × 10^−6^), candidate SNPs were screened from the exposure variables. The linkage disequilibrium (LD) among the SNPs of each exposure variable was calculated using PLINK aggregation (Slatkin, 2008). Among them, the SNPs with *r* ^2^ > 0.001 and base physical distance <10,000 kb were removed, and the SNP with the lowest *p*-value was retained. Candidate SNPs were matched to the GWAS data on the outcome variables based on the chromosome and location. To further evaluate the strength of each instrumental variable, we calculated the F-statistic using the following formula:

$$F=R^{2}\frac{\left(N-2\right)}{\left(1-R^{2}\right)},$$

where N is the sample size and *R* ^2^ is genetic variation; the SNPs with F < 10 were excluded to guarantee that the instrumental variables have sufficient validity and instrumental strength. The genetic variation *R* ^2^ was calculated using the following formula:

$$R^{2}=2\times EAF\times \left(1-EAF\right)\times \beta^{2},$$

where β is the allele effect value and EAF is the effect allele frequency. Simultaneously, since some row data do not have effect allele frequency. The F-statistic could also be obtained using the Cragg–Donald statistic, F = β_exposure_^2^/SE_exposure_^2^ equation (Cragg and Donald, 1993). Subsequently, PhenoScannerV2 (http://www.phenoscanner.medschl.cam.ac.uk/) was used to exclude outcome- and confounder-associated SNPs. In this study, IBS, hypothyroidism, opioid abuse, Parkinson’s disease, and aging-related traits including the telomere length and problems related to life-management difficulty were regarded as confounders (Jaul and Barron, 2017; Stocchi and Torti, 2017; Bharucha and Wald, 2019; Zhang et al., 2022). Finally, we harmonized the exposure and outcome datasets to guarantee that the effect alleles belonged to the same alleles, and SNPs palindromic with intermediate allele frequencies were removed.

### 2.4 Statistical analysis

We primarily studied the effect of each GF on constipation using univariate two-sample MR. Inverse-variance weighted (IVW), MR–Egger regression, weight median, simple mode, and weight mode methods were used to determine genetic associations. The IVW method was the main analysis method because it presupposes that all genetic variants satisfy the three assumptions of IVs and provides the most accurate estimates in the absence of horizontal pleiotropy and heterogeneity (Emdin et al., 2017). The causal effect was estimated based on the influence of the regression effect coefficient on the exposure effect coefficient. Considering the potential bias from IV-level pleiotropy, we supplemented the IVW method with MR–Egger regression and the weighted median method. MR–Egger regression is largely similar to the IVW method, except that its regression model includes an intercept to reflect horizontal pleiotropy (Burgess and Thompson, 2017). Compared with IVW and MR–Egger regression, the weighted median method is more robust to invalid IVs (Bowden et al., 2016).

### 2.5 Sensitivity test

Cochran’s Q test was used to test the heterogeneity of the associations using the MR–Egger and IVW methods, with *p* < 0.05 indicating heterogeneity. Then, the Egger intercept and Mendelian Randomization Pleiotropy RESidual Sum and Outlier (MR-PRESSO) tests were used to test horizontal pleiotropy. The MR funnel plot also exhibited horizontal pleiotropy due to the symmetry of the SNPs. The MR-PRESSO test detects the significant outlier in MR analysis and re-evaluates causal effects after the removal of the detected outlier SNPs. The MR-PRESSO distortion test was used to test for significant distortion in causal estimates before and after outlier removal (Verbanck et al., 2018). R software (version 4.2.0), with the R packages “two-sample MR” and “MR-PRESSO,” was used to conduct all the statistical analyses. In this study, statistical significance was set at *p* < 0.05.

## 3 Results

### 3.1 Instrumental variable selection

Since several exposure variables were included in this study, only the variables with significant results are shown. The independent SNPs strongly associated with exposure were screened as candidate IVs. Then, PhenoScannerV2 was used to exclude the confounder- and outcome-associated SNPs; however, no SNPs were eliminated. Then, the exposure and outcome datasets were harmonized, and the SNPs palindromic with intermediate allele frequencies were removed. After strict program screening, the number of the included IVs ranged from 3 to 19 (Supplementary Table S1). The F-statistics of all the IVs are higher than 10, indicating that these IVs are all effective and that the weak instrumental variable bias does not affect the causal inference results of the MR analysis in this study (Supplementary Table S2).

### 3.2 Causal associations between GFs and constipation

**FIGURE 2:**
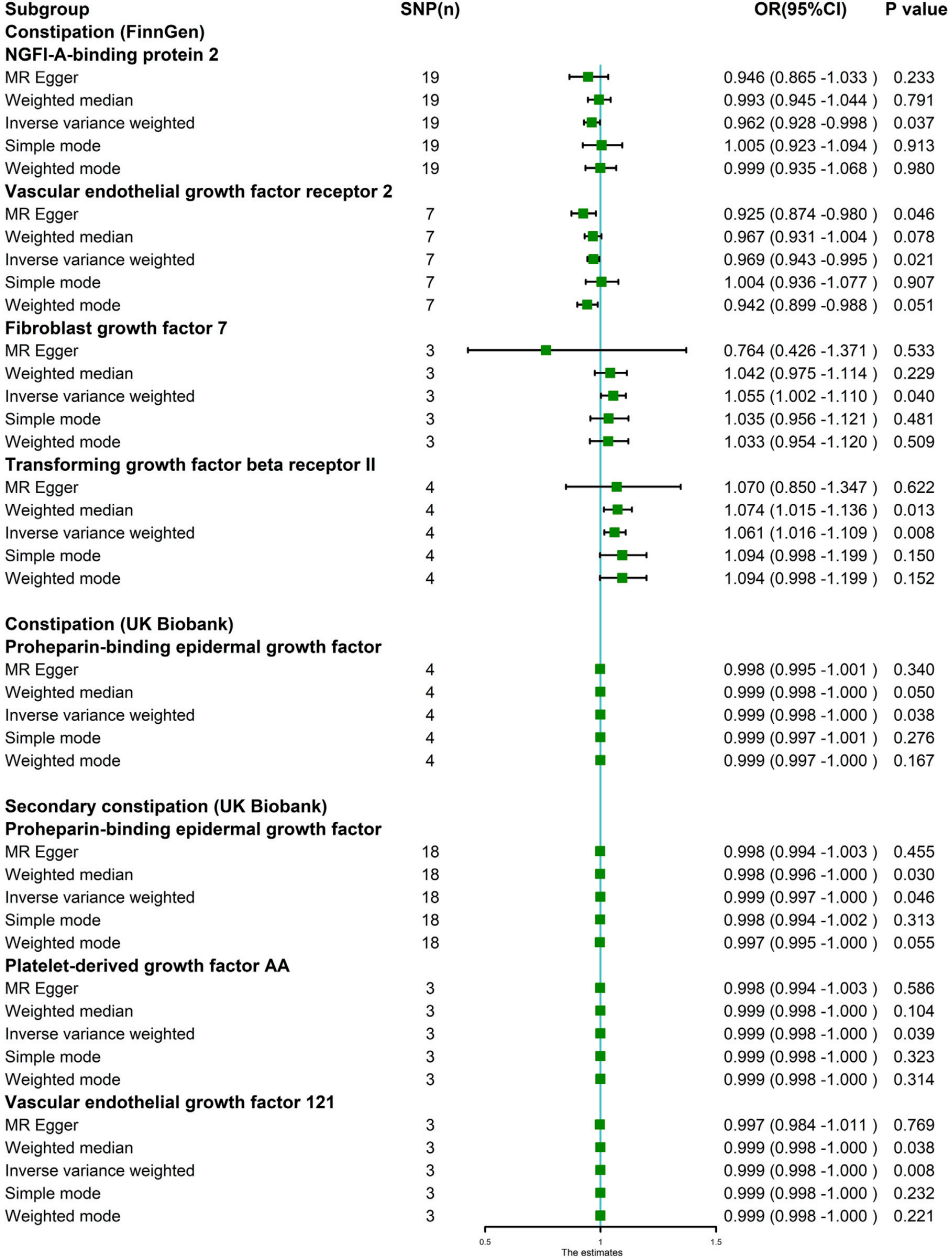
MR estimates assessing the significant causal effect of growth factors on constipation. Five MR methods were used to obtain the estimate between growth factors on constipation. The IVW method was regarded as the major method in this figure. IVW analysis revealed that in the FinnGen project, NGFI-A-binding protein 2 and vascular endothelial growth factor receptor 2 levels were inversely associated with constipation. Fibroblast growth factor 7 and transforming growth factor beta receptor II levels were positively associated with constipation. Simultaneously, in the UK Biobank GWAS dataset, proheparin-binding epidermal growth factor, platelet-derived growth factor AA, and vascular endothelial growth factor_121_ levels were inversely associated with constipation risk.

**FIGURE 3:**
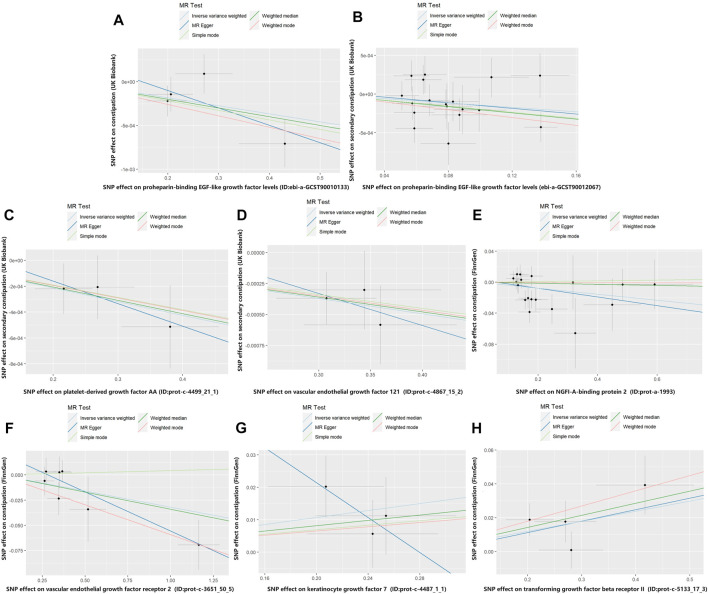
Scatter plot of genetic correlations for growth factors on constipation. The slope of the line represents the causal effect of each method. **(A)** Scatter plot of genetic correlations for proheparin-binding EGF-like growth factor levels on constipation (UK Biobank). **(B)** Scatter plot of genetic correlations for proheparin-binding EGF-like growth factor levels on secondary constipation (UK Biobank). **(C)** Scatter plot of genetic correlations for platelet-derived growth factor AA on secondary constipation (UK Biobank). **(D)** Scatter plot of genetic correlations for vascular endothelial growth factor_121_ on secondary constipation (UK Biobank). **(E)** Scatter plot of genetic correlations for NGFI-A-binding protein 2 on constipation (FinnGen). **(F)** Scatter plot of genetic correlations for vascular endothelial growth factor receptor 2 on constipation (FinnGen). **(G)** Scatter plot of genetic correlations for keratinocyte growth factor 7 on constipation (FinnGen). **(H)** Scatter plot of genetic correlations for transforming growth factor beta receptor II on constipation (FinnGen).

**FIGURE 4:**
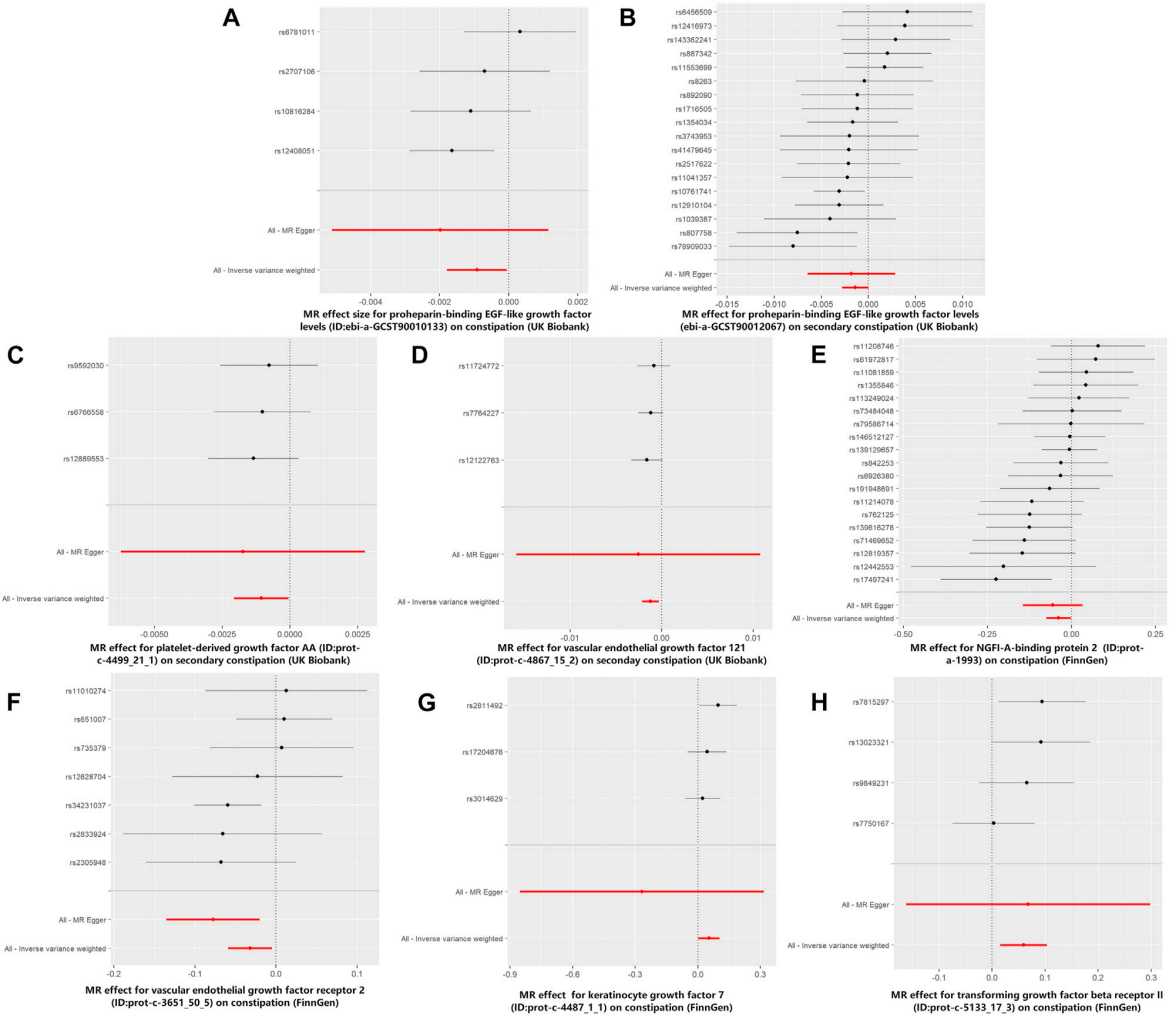
Forest plots for MR analyses of the causal effect of growth factors on constipation. The red and black dots/bars in the forest plots represent causal estimates of the growth factors on constipation. **(A)** Forest plot of the causal effects of proheparin-binding EGF-like growth factor levels on constipation (UK Biobank). **(B)** Forest plot of the causal effects of proheparin-binding EGF-like growth factor levels on secondary constipation (UK Biobank). **(C)** Forest plot of the causal effects of platelet-derived growth factor AA on secondary constipation (UK Biobank). **(D)** Forest plot of the causal effects of vascular endothelial growth factor_121_ on secondary constipation (UK Biobank). **(E)** Forest plot of the causal effects of NGFI-A-binding protein 2 on constipation (FinnGen). **(F)** Forest plot of the causal effects of vascular endothelial growth factor receptor 2 on constipation (FinnGen). **(G)** Forest plot of the causal effects of keratinocyte growth factor 7 on constipation (FinnGen). **(H)** Forest plot of the causal effects of transforming growth factor beta receptor II on constipation (FinnGen).

In the MR analysis using the outcome data obtained from FinnGen, the IVW analysis genetically predicted that NGFI-A-binding protein 2 (OR = 0.962, 95% CI: 95% CI: 0.928–0.998, and *p* = 0.037) and vascular EGF (VEGF) receptor 2 (OR = 0.969, 95%CI:95% CI: 0.943–0.995, and *p* = 0.021) were inversely associated with constipation. Moreover, the IVW results showed that keratinocyte growth factor 7 (OR = 1.055, 95%CI: 95% CI: 1.002–1.110, and *p* = 0.040) and TGF beta receptor 2 (TGF-βRII; OR = 1.061, 95%CI: 95% CI: 1.016–1.109, and *p* = 0.008) levels were positively associated with constipation. Simultaneously, in the MR analysis using the outcome data obtained from the UK Biobank, the IVW analysis predicted that proheparin-binding EGF (proHB-EGF constipation: OR = 0.999, 95%CI: 95% CI: 0.998–1.000, and *p* = 0.038; secondary constipation: OR = 0.999, 95%CI: 95% CI: 0.997–1.000, and *p* = 0.046), platelet-derived growth factor AA (PDGF-AA; OR = 0.999, 95%CI: 95% CI: 0.998–1.000, and *p* = 0.039), and vascular endothelial growth factor_121_ (VEGF_121_; OR = 0.999, 95%CI: 95% CI: 0.998–1.000, and *p* = 0.008) levels were inversely associated with constipation. However, the OR values of these associations are very close to 1, and we further believe that these growth factors have a suggestive protective effect on constipation. Further studies are needed to verify these associations. Figure 2 shows the genetic estimates obtained using the five MR methods, and the scatter plots (Figure 3) and forest plots (Figure 4) show the results more intuitively. The abscissa of the scatter plots is the causal effect of the IVs on the outcome, and the ordinate is the causal effect of the IVs on the exposure. The slopes of the lines represent the causal effect of each method. Simultaneously, the forest plots show the effect of individual SNPs on the causal effect between GFs and constipation.

### 3.3 Sensitivity and heterogeneity analysis

**FIGURE 5:**
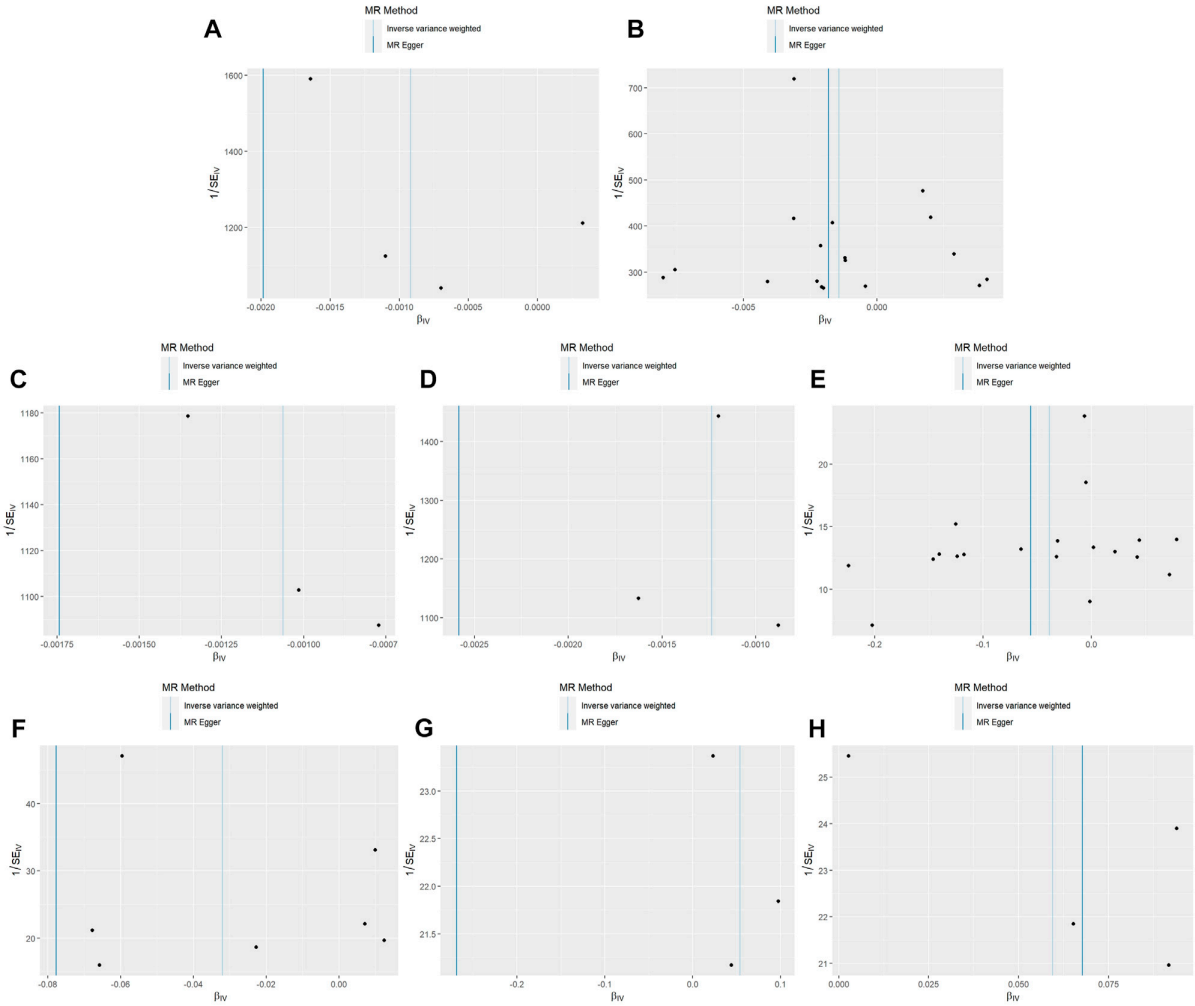
Funnel plots for MR analyses of the causal effect of growth factors on constipation. Funnel plot showing the effect of signal SNPs on the association between growth factors and constipation. **(A)** Funnel plot of the causal effects of proheparin-binding EGF-like growth factor levels on constipation (UK Biobank). **(B)** Funnel plot of the causal effects of proheparin-binding EGF-like growth factor levels on secondary constipation (UK Biobank). **(C)** Funnel plot of the causal effects of platelet-derived growth factor AA on secondary constipation (UK Biobank). **(D)** Funnel plot of the causal effects of vascular endothelial growth factor_121_ on secondary constipation (UK Biobank). **(E)** Funnel plot of the causal effects of NGFI-A-binding protein 2 on constipation (FinnGen). **(F)** Funnel plot of the causal effects of vascular endothelial growth factor receptor 2 on constipation (FinnGen). **(G)** Funnel plot of the causal effects of keratinocyte growth factor 7 on constipation (FinnGen). **(H)** Funnel plot of the causal effects of transforming growth factor beta receptor II on constipation (FinnGen).

Cochran’s Q-value test was performed to identify the heterogeneity in these results. Both the IVW and MR–Egger methods showed that heterogeneity did not exist in these associations (*p* > 0.05). Table 1 shows the heterogeneity results obtained using MR–Egger. The Egger intercept test showed no evidence of horizontal pleiotropy in these results (*p* > 0.05). The funnel plots show that the distribution of SNPs was symmetrical (Figure 5). Moreover, MR-PRESSO did not detect significant outliers or horizontal pleiotropy in these results, except for the association between NGFI-A-binding protein 2 and secondary constipation, because the IVs of this association were insufficient.

**TABLE 1:** Sensitivity test.

Exposure	Outcome	Cochran’s Q test		Egger intercept test		MR-PRESSO	
		Q-Egger	*p*-value	Egger intercept	*p*-value	*p*-value of the global test	*p*-value of the distortion test
NAB2	Constipation (Finn)	22.115	0.180	0.003	0.679	0.261	NA
VEGF sR2	Constipation (Finn)	2.914	0.713	0.022	0.138	0.376	NA
FGF7	Constipation (Finn)	0.282	0.595	0.075	0.474	NA	NA
TGF-β R II	Constipation (Finn)	3.230	0.199	−0.002	0.949	0.414	NA
proHB-EGF	Secondary constipation	20.893	0.183	<0.001	0.863	0.260	NA
PDGF-AA	Secondary constipation	0.127	0.722	0.001	0.812	NA	NA
VEGF121	Secondary constipation	0.308	0.579	<0.001	0.875	0.957	NA
pro-HB-EGF	Constipation	2.976	0.226	<0.001	0.556	0.127	NA

Note: NAB2: NGFI-A-binding protein 2; VEGF sR2: vascular endothelial growth factor receptor 2; FGF7: fibroblast growth factor 7; TGF-β R II: transforming growth factor beta receptor II; proHB-EGF: proheparin-binding epidermal growth factor; PDGF-AA: platelet-derived growth factor AA; VEGF121: vascular endothelial growth factor_121_; NA: not applicable.

## 4 Discussion

This is the first two-sample MR to explore the causal associations of 77 GFs (including these receptors) with primary and secondary constipation. In this study, GFs and their receptors, including pro-HB-EGF, VEGF_121_, PDGF-AA, NGFI-A-binding protein 2, FGF7, IGF-1, TGF-βRII, and VEGF sR2, causally associated with constipation were screened; the causal association between pro-HB-EGF and TGF-βRII and constipation was the most significant (*p* < 0.01). Furthermore, pro-HB-EGF was negatively associated with constipation, and TGF-βRII was positively associated with constipation, which indicates that TGF-β, its related signal pathway, and pro-HB-EGF may affect the development of constipation.

TGF-β is a cytokine present in all cells and regulates cell proliferation and differentiation. It has various biological functions, including inhibiting proliferation, promoting apoptosis, and regulating immunity (Larson et al., 2020; Tzavlaki and Moustakas, 2020). TGF-β can be structurally categorized into three subtypes: TGF-β1, TGF-β2, and TGF-β3. TGF-β1 is the most abundant subtype in mammals and closely related to intestinal immune diseases (Ihara et al., 2017). This study genetically predicated that TGF-βRII was positively associated with constipation. Activated TGF-β binds to TGF-βRII, which, in turn, forms a complex with TGF-βRI. The resulting TGF-β receptor complex activates intracellular signaling through both Smad-dependent canonical and Smad-independent non-canonical pathways (Travis and Sheppard, 2014). A cross-sectional study has revealed significantly higher TGF-β levels in patients with IBD than those in the normal population (Babyatsky et al., 1996). Moreover, Del Zotto et al. (2003) reported that TGF-β1 production in intestinal mucosal lamina propria lymphocytes is significantly higher in patients with ulcerative colitis than that in healthy participants (Del Zotto et al., 2003). Simultaneously, active TGF-β levels in narrow intestinal muscle obtained from a surgically resected ileum of patients with Crohn’s disease were higher than those in adjacent normal intestinal tissue (Li et al., 2013a). This shows that TGF-β and TGF-β-related signaling pathways may be risk factors for IBD. Constipation is a clinical manifestation of IBD. We speculate that TGF-β and its related signaling pathway may promote IBD, resulting in symptoms of constipation. Furthermore, TGF-β may be associated with the transmission function of the colon. Zhang et al. (2022) confirmed that TGF-β/Smad signaling pathway activation can induce epithelial–mesenchymal transition, causing abnormal ICC distribution and dysfunction in the diseased colon, ultimately promoting the occurrence and development of slow transit constipation.

HB-EGF is a member of the EGF family with strong heparin-binding ability. It primarily promotes mitosis of various cells such as fibroblasts, smooth muscle cells, and epithelial cells (Liao et al., 2013). We found that proHB-EGF is inversely associated with constipation. Current studies generally believe that high HB-EGF expression reduces inflammation-related damage to the intestinal mucosa and promotes intestinal epithelial cell repair, proliferation, and regeneration (Feng et al., 2005). Zhou et al. (2022) found that HB-EGF significantly reduces TNF-induced intestinal epithelial cell death *in vitro* and also synergizes with ErbB1 signaling to prevent cell death (Zhou et al., 2022). In addition, an animal experiment using HB-EGF confirmed that intraluminal HB-EGF injection significantly reduces intestinal tissue damage, pro-inflammatory cytokine expression, myeloperoxidase activity, malondialdehyde level, and apoptosis index in mice (Liao et al., 2013), suggesting that HB-EGF may protect against intestinal inflammatory diseases, which is also consistent with our results. Therefore, we speculate that HB-EFG was negatively associated with constipation because it is a protective factor for IBD.

The FGF family signals through FGF receptor tyrosine kinases to regulate a broad range of biological processes during development and adulthood (Beenken and Mohammadi, 2009). We showed that FGF7 in human plasma is positively associated with constipation. FGF7 is also called keratinocyte growth factor (KGF); Visco et al. (2009) confirmed that KGF and its receptors directly influence intestinal epithelial growth and differentiation regulation (Visco et al., 2009). It prevents intestinal mucosal barrier disruption; however, KGF overexpression can cause abnormal intestinal epithelial cell proliferation and may lead to colorectal cancer (Finch and Rubin, 2004; Shantha Kumara et al., 2021). Therefore, intestinal epithelial cell hyperproliferation may explain the association between FGF7 and constipation.

The etiology of constipation is complex and associated with colonic sensorimotor disturbances, pelvic floor dysfunction, changes in colon physiology, and various colonic diseases (Bharucha and Lacy, 2020). We used two-sample MR to screen several GFs related to constipation, which provides a direction for further research on their role in intestinal physiological functions and pathological processes. Since constipation is a common symptom of gastrointestinal diseases, exploring the GFs related to constipation can provide evidence for the role of various GFs in the pathogenesis of other gastrointestinal diseases, such as TGF-β and HB-EFG in IBS, and FGF7 in intestinal epithelial cell hyperproliferation. The MR method minimizes the possibility of reverse causation and potential confounding factors, and the results are reliable and robust due to the use of different sensitivity tests. Furthermore, all SNPs used as IVs were screened rigorously through their significance and independence, and the F-value of all the IVs was >20, implying that there is little chance of weak instrumental variable bias.

This study had several shortcomings. First, due to the limitations of GWAS data, we could not investigate the relationship between GFs and specific constipation types or constipation caused by different diseases; simultaneously, considering that the current sample size of GFs is relatively small, their effect strength will be limited. We look forward to more GWAS summary data to verify the genetic causality between GFs and constipation. Second, horizontal pleiotropy was inevitable in the analysis; however, we performed weighted median analysis, MR–Egger regression, and used various sensitivity tests, including Cochran’s Q, Egger intercept, and MR-PRESSO tests. Third, the summary GWAS data used in this study were collected from only European individuals, and further studies should be conducted to generalize these findings to other populations. Fourth, in two-sample MR studies, sample overlap will lead to biased effect estimates, and researchers can only control this error by replacing the GWAS summary data on related traits (Burgess et al., 2016). Therefore, sample overlap remains one of the main potential limitations of such MR studies. However, the GWAS summary data of GFs in this study were primarily from INTERVAL and studies conducted by Folkersen et al. (2020) and Suhre et al. (2017). The GWAS summary data of constipation were from FinnGen and UK Biobank. As exposures and outcomes were independent in the summary data, we considered the degree of sample overlap to be acceptable in this study; furthermore, because the SNP-associated data used in our study are from studies analyzing populations of European ancestry alone, this study can avoid population stratification bias. Finally, the MR approach can only provide genetic evidence for a causal association between GFs and constipation, and further independent validation analyses of these causal associations are needed in the future.

## 5 Conclusion

In this study, two-sample MR was used to systematically evaluate the causal effects of 77 GFs. The results suggest that TGF-β and related signaling pathways may be risk factors for constipation, while HB-EGF protects against constipation. Although further studies are needed to confirm these results, they still provide clues for a comprehensive analysis of the molecular mechanism and signaling pathways of constipation and provide new ideas for targeted constipation treatment.

## Data Availability

The original contributions presented in the study are included in the article/Supplementary Material; further inquiries can be directed to the corresponding author.
